# Gold nanoparticles improve metabolic profile of mice fed a high-fat diet

**DOI:** 10.1186/s12951-018-0338-1

**Published:** 2018-02-06

**Authors:** Hui Chen, Jane P. M. Ng, Yi Tan, Kristine McGrath, David P. Bishop, Brian Oliver, Yik Lung Chan, Michael B. Cortie, Bruce K. Milthorpe, Stella M. Valenzuela

**Affiliations:** 1 Molecular Biosciences Team, School of Life Sciences, Faculty of Science, University of Technology Sydney, Sydney, NSW 2007 Australia; 2 Centre for Health Technology, University of Technology Sydney, Sydney, NSW 2007 Australia; 3 School of Mathematical and Physical Sciences, Faculty of Science, University of Technology Sydney, Sydney, NSW 2007 Australia; 4 Institute for Nanoscale Technology, University of Technology Sydney, Sydney, NSW 2007 Australia

**Keywords:** Obesity, Gold nanoparticles, Inflammation, Lipid metabolism, Glucose intolerance

## Abstract

**Background:**

Obesity is a high risk for multiple metabolic disorders due to excessive influx of energy, glucose and lipid, often from a western based diet. Low-grade inflammation plays a key role in the progression of such metabolic disorders. The anti-inflammatory property of gold compounds has been used in treating rheumatoid arthritis in the clinic. Previously we found that pure gold nanoparticles (AuNPs, 21 nm) also possess anti-inflammatory effects on the retroperitoneal fat tissue following intraperitoneal injection, by downregulating tumor necrosis factor (TNF) α. However, whether such an effect can change the risk of metabolic disorders in the obese has not been well studied. The study employed C57BL/6 mice fed a pellet high fat diet (HFD, 43% as fat) that were treated daily with AuNPs [low (HFD-LAu) or high (HFD-HAu) dose] via intraperitoneal injection for 9 weeks. In the in vitro study, RAW264.7 macrophages and 3T3-L1 adipocytes were cultured with low and high concentrations of AuNPs alone or together.

**Results:**

The HFD-fed mice showed a significant increase in fat mass, glucose intolerance, dyslipidemia, and liver steatosis. The HFD-LAu group showed an 8% reduction in body weight, ameliorated hyperlipidemia, and normal glucose tolerance; while the HFD-HAu group had a 5% reduction in body weight with significant improvement in their glucose intolerance and hyperlipidemia. The underlying mechanism may be attributed to a reduction in adipose and hepatic local proinflammatory cytokine production, e.g. TNFα. In vitro studies of co-cultured murine RAW264.7 macrophage and 3T3-L1 adipocytes supported this proposed mechanism.

**Conclusion:**

AuNPs demonstrate a promising profile for potential management of obesity related glucose and lipid disorders and are useful as a research tool for the study of biological mechanisms.

**Electronic supplementary material:**

The online version of this article (10.1186/s12951-018-0338-1) contains supplementary material, which is available to authorized users.

## Background

Obesity is an important risk factor for multiple metabolic disorders, including glucose intolerance and hyperlipidemia. The current global surge in obesity has seen a staggering 800% increase in demand for weight-loss surgical procedures over the last decade, as a means of controlling these metabolic disorders [1]. This increase is also driven by the disappointingly low success rate of weight-loss medications and interventions, as well as the difficulties faced by individuals trying to maintain ideal body weight following initial weight loss. For example, in a recent trial, the latest approved injectable weight loss medication, Liraglutide (Saxenda) has been shown to induce ~ 6% of total body weight loss after 56 weeks of treatment [2]. However, this weight loss effect required daily adherence to a strict low-caloric diet and ongoing support by dieticians, making its implementation difficult to achieve outside of a closely controlled environment [2]. Therefore, there still remains an urgent and growing need for effective strategies to deal with the global obesity pandemic. Herein, we present intriguing evidence that gold nanoparticles (AuNPs) may serve as a novel therapeutic agent in the treatment and control of obesity and its related blood glucose and lipid disorders.

There is already historical precedence for the use and application of bulk gold and gold salts within clinical practice [3]. It is now becoming evident that AuNPs share similar therapeutic potentials [4]. Nanomaterials have been widely applied in medicine as biochemical sensors, contrast agents in imaging, and drug delivery vehicles revolutionizing current disease treatment and diagnosis [4]. However, the function and toxicity of AuNPs differ subtantially depending on the size and shape with AuNPs larger than 15 nm comparatively nontoxic [5].

Previously, we injected unmodified spherical AuNPs of 21 nm diameter into chow-fed lean mice [6]. The AuNPs accumulated rapidly in the abdominal fat tissue after a single intraperitoneal (IP) injection. AuNP-treated mice showed significant reduction in abdominal fat mass compared to non-treated control mice, along with reduced mRNA expression of the pro-inflammatory cytokines, tumor necrosis factor (TNF)-α, in the abdominal fat tissue [6]. This is of great interest, as TNF-α has been frequently linked to the comorbidities related to obesity [7]. In chronic obesity, excess triglyceride storage in the fat tissue can up-regulate adipose triglyceride lipase (ATGL) to increase basal lipolysis [8]. Consequently, adipose tissue macrophage (ATM) infiltration and accumulation into the fat tissue is also increased, which promotes inflammatory responses in the adipose tissue by directly engaging toll-like receptors (TLR) to induce production of cytokines, such as TNFα [9]. For these reasons, TNFα expression is positively correlated with body mass index, hyperlipidemia, insulin resistance, and glucose intolerance [10, 11]. Either reducing ATM recruitment or inhibiting ATM cytokine release can lead to fat loss and improved insulin sensitivity in obese mice [9, 12]. This highlights the essential roles of ATM-related cytokines in the development of metabolic disorders in obesity. The down-regulation of pro-inflammatory cytokines in our previous study was linked to reduced ATM activity, rather than reduced cell number [6]. In addition, the abdominal fat loss induced AuNP treatment was also of interest for its potential to treat obesity.

Although the anti-inflammatory property of bulk gold and AuNPs has been clinically used for treating rheumatoid arthritis [3], the injectable AuNP preparation has not been reported for managing adiposity and metabolic disorders in obesity. Therefore, in the current study we IP injected AuNPs into mice fed a high-fat diet (HFD) for 9 weeks to examine the effect on fat accumulation and obesity related metabolic disorders. In addition, our in vitro studies investigated the direct impact of the AuNPs on adipocyte and macrophage interactions. The knowledge gained from this study will serve to inspire new, original and more effective therapeutic approaches that involve direct targeting of intracellular pathways in adipocytes and/or macrophage cells.

## Methods

### Animal experiments

Male C57Bl/6 mice (8 weeks, Animal Resource Centre, WA, Australia) were then randomly divided into 4 groups (*n* = 20, Table 1). Control group (Chow-C) were fed chow (Gordon’s Specialty Stockfeeds, NSW, Australia) and injected with vehicle; HFD group (HFD-C) was fed a HFD (20 kJ/g, 43% fat, Cat. SF03-020, Specialty Feeds, WA, Australia) ad libitum and injected with vehicle; low dose AuNP (HFD-LAu) group fed a HFD and received AuNP (0.785 μg Au/g, IP); and high dose AuNP (HFD-HAu) group fed a HFD and received AuNP (7.85 μg Au/g, IP) determined according to our previous study [6]. The HFD has been repeatedly used to induce obesity in rodents by us [13–18]. The chow-fed mice treated with AuNP was not adopted in this study as we have shown the fat loss effect in lean mice [6] and lean humans rarely requires weight loss treatment. AuNPs were prepared as previously described [6], and injection was performed at 10 am daily for 9 weeks. Food intake and body weight was monitored weekly. IP glucose tolerance test (IPGTT) was performed at 8 weeks in randomly selected mice from each group as previously described [15]. The area under the curve (AUC) of glucose levels was calculated for each mouse. Tissues were harvested at 9 weeks after Pentothal (0.1 mg/g, IP, Abbott Diagnostics, NSW, Australia) anesthesia. Blood glucose was measured (Accu-Check^®^, Roche, CA, USA) and plasma was stored at − 80 °C. Heart, spleen, kidneys, liver, and abdominal fat pads were weighed and either fixed in 10% formalin or snap frozen in liquid nitrogen and stored at − 80 °C. All tissue analysis was performed in a blind manner and the results were only identified before data analysis.

**Table 1:** Anthropometry of mice after 9 weeks of HFD and AuNP treatments

	Chow-C	HFD-C	HFD-LAu	HFD-HAu
Body weight initial (g)	20.2 ± 0.3	20.2 ± 0.3	20.3 ± 0.2	20.2 ± 0.3
Body weight final (g)	27.7 ± 0.3	37.5 ± 1.2*	34.5 ± 0.8*^†^	35.8 ± 0.6*
Energy intake (kJ/day)	44.9 ± 0.6	48.4 ± 1.4	50.5 ± 1.4*	52.3 ± 1.4*^†^
Heart (g)	0.135 ± 0.002	0.145 ± 0.004	0.138 ± 0.004	0.141 ± 0.004
Heart (%)	0.49 ± 0.01	0.40 ± 0.01*	0.39 ± 0.02*	0.40 ± 0.01*
Kidney (g)	0.165 ± 0.004	0.192 ± 0.005*	0.179 ± 0.004*	0.188 ± 0.005*
Kidney (%)	0.59 ± 0.01	0.51 ± 0.02*	0.49 ± 0.02*	0.53 ± 0.02*
Liver (g)	1.34 ± 0.03	1.86 ± 0.12*	1.49 ± 0.03*^†^	1.54 ± 0.04*^†^
Liver (%)	4.86 ± 0.08	5.03 ± 0.12	4.27 ± 0.07*^†^	4.30 ± 0.10*^†^
Retroperitoneal fat (g)	0.097 ± 0.006	0.761 ± 0.054*	0.652 ± 0.044*	0.616 ± 0.048*^†^
Retroperitoneal fat (%)	0.35 ± 0.02	2.01 ± 0.10*	1.86 ± 0.11*	1.77 ± 0.09*^†^
Mesenteric fat (g)	0.372 ± 0.018	0.939 ± 0.075*	0.741 ± 0.060*^†^	0.691 ± 0.029*^†^
Mesenteric fat (%)	1.35 ± 0.06	2.46 ± 0.14*	2.07 ± 0.28*^†^	1.97 ± 0.07*^†^
Epididymal fat (g)	0.405 ± 0.013	2.029 ± 0.19*	1.77 ± 0.12*	1.80 ± 0.09*
Epididymal fat (%)	1.43 ± 0.06	5.38 ± 0.35*	5.07 ± 0.14*	4.98 ± 0.19*
Plasma insulin (ng/ml)	1.05 ± 0.12	1.09 ± 0.12	1.44 ± 0.21*^†^	1.11 ± 0.05
Plasma NEFA (nm)	2.54 ± 0.16	4.51 ± 0.54*	2.9 ± 0.24^†^	3.14 ± 0.35^†^
Plasma cholesterol (mm)	6.24 ± 0.31	11.14 ± 0.36*	9.40 ± 0.29*^†^	9.46 ± 0.41*^†^
Plasma triglyceride (mm)	0.75 ± 0.05	0.86 ± 0.05	0.67 ± 0.04^†^	0.69 ± 0.05^†^
Liver triglyceride (mm/mg)	0.023 ± 0.005	0.124 ± 0.019*	0.139 ± 0.007*	0.145 ± 0.010*
Plasma ALT (U/L)	17.3 ± 6.2	55.9 ± 13*	25.5 ± 6.9^†^	26.3 ± 2.4^†^
Plasma AST (U/L)	6.33 ± 2.16	31.4 ± 7.1*	16.9 ± 3.9^†^	12.1 ± 2.2^†^

Data are expressed in mean ± S.E.M. Data were analyzed using one-way ANOVA, followed by post hoc Bonferroni tests

*n* = 20/group for anthropometry markers; *n* = 8/group for plasma insulin, NEFA, cholesterol, triglyceride, and liver triglyceride concentrations; *n* = 6/group for plasma ALT and AST concentration

* *P* < *0.05* vs. Chow-C; ^†^ *P* < *0.05* vs. HFD-C

### In vitro experiments and gold nanoparticle synthesis

Details are in Additional file 1.

#### Biochemical analysis

Plasma and cell supernatant triglycerides were measured using an in house assay using glycerol standards (Sigma-Aldrich, MO, USA) and triglyceride reagent (Roche Diagnostics, NJ, USA) [13, 15]. Nonesterified free fatty acid (NEFA) was measured using a NEFA kit (WAKO, Osaka, Japan) [19]. Plasma alanine aminotransferase (ALT) and aspartate aminotransferase (AST) were measured using commercial kits (Dialab Ltd., Vienna, Austria) as an indicator of liver cell damage. Plasma cholesterol concentration was measured using the Cholesterol CHOD-PAP with ATCS kit (Dialab Ltd., Vienna, Austria).

#### Quantitative real-time PCR

Total RNA was isolated (*n* = 5–10 randomly selected from each group, cells *n* = 8–10) using TRI reagent (Sigma-Aldrich, MO, USA). First-strand cDNA was synthesized using M-MLV Reverse Transcriptase, RNase H Minus, Point Mutant Kit (Promega, WI, USA) [20, 21]. Pre-optimized TaqMan^®^ probe/primers (Additional file 1: Table S1, Life Technologies, CA, USA) and SYBR^®^ Green premiers (Additional file 1: Table S2, Bio-Rad, CA, USA) [22] were used for the real-time PCR (Eppendorf Realplex^2^, Hamburg, Germany). The genes of interest were normalized against the housekeeping gene 18s rRNA (Additional file 1: Table S1). The average value of the control was assigned as the calibrator, against which all other samples are expressed as a fold difference.

#### Immunohistochemistry

Formalin fixed liver and abdominal fat samples (n = 5) were embedded in paraffin and sectioned (4 µm). To explore F4/80 positive cells sections were incubated with a rabbit anti-mouse F4/80 (Abcam, Cambridge, UK) primary antibodies, and visualised using the horseradish peroxidase anti-rabbit Envision system (Dako Cytochemistry, Tokyo, Japan). The sections were then counterstained with haematoxylin. Three images from each section were captured and used for analysis. The F4/80-expressing cells were counted and expressed as the percentage of total cell number for a sample total number of nuclei and the number of nuclei of for each field.

#### Statistical analysis

The results were expressed as mean ± standard error of the mean (S.E.M). The data was analyzed using one-way ANOVA, followed by post hoc Bonferroni tests (Statistica 10. StatSoft Inc. OK, USA), if normally distributed. If the data was not normally distributed, they were log transformed to achieve normality of distribution before they were analyzed. The glucose levels during IPGTT were analyzed using one-way ANOVA with repeat measures followed by post hoc Bonferroni test. P < 0.05 was considered significant.

## Results

### In vivo animal study

#### Anthropometry

**Fig. 1:**
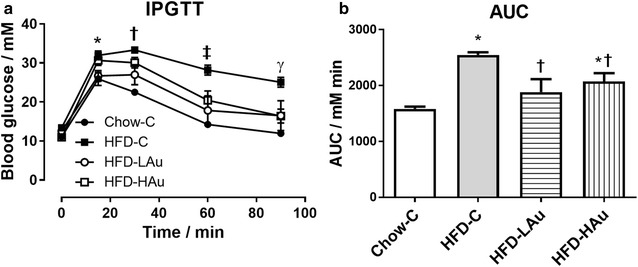
**a** intraperitoneal glucose tolerance test (IPGTT, glucose 2 g/kg), **b** area under the curve (AUC) of the (**a**), at 8 weeks of treatment. Data are expressed in mean ± S.E.M. IPGTT difference in (**a**) were analyzed using one-way ANOVA with repeat measures followed by post hoc Bonferroni test. *P < 0.05, Chow-C and HFD-LAu vs. HFD-C at 15 min; ^†^P < 0.05, Chow-C and HFD-LAu vs. HFD-C at 30 min; ^‡^P < 0.05, Chow-C, HFD-LAu, and HFD-HAu vs. HFD-C at 60 min; ^γ^P < 0.05, Chow-C, HFD-LAu, and HFD-HAu vs. HFD-C at 90 min. AUC difference in (**b**) were analyzed using one-way ANOVA followed by post hoc Bonferroni test. *P < *0.05* vs. Chow-C group; ^†^*P* < *0.05* vs. HFD-C group; *n* = 6

Four groups of mice started with similar body weight (Table 1). At 9 weeks post-treatment, the HFD-fed group (HFD-C) was 35% heavier than the Control (Chow-C) group, with significantly increased organ and fat masses, as well as blood lipid cholesterol and NEFA concentrations (*P* < 0.05, Table 1). Adipocyte size was more than doubled in the HFD-C group (*P* < 0.01 *vs.* Chow-C, Additional file 1: Figure S1). Plasma ALT and AST levels were ~ 3 and 5 times higher in the HFD-C group (*P* < 0.05 vs. Chow-C, Table 1). Blood glucose levels during IPGTT were also significantly higher in the HFD-C group than the Chow-C group, from 15 to 90 min post glucose injection (*P* < 0.05, Fig. 1a), with 60% greater AUC value (*P* < 0.05, Fig. 1b).

The two groups of mice treated with AuNPs consumed more energy than the Chow-C and HFD-C groups (*P* < 0.05, Table 1). However, the body weights of the HFD-LAu and HFD-HAu groups were 8 and 5% smaller than the HFD-C mice, respectively (*P* < 0.05). Smaller fat masses were observed in the AuNP-treated mice (*P* < 0.05 retroperitoneal, HFD-C vs. HFD-HAu; mesenteric, HFD-C vs. HFD-LAu and HFD-HAu, Table 1). However, the fat cell size was larger in the HFD-LAu group, but smaller in the HFD-HAu group (both *P* < 0.01 vs. HFD-C, Additional file 1: Figure S1). Both AuNP-treated groups had significantly lower blood lipid levels than the HFD-C group (*P* < 0.05) with nearly normalized liver AST and ALT levels (*P* < 0.05, Table 1). These results suggest a lipid lowering effect by the AuNPs and long-term safety and benefit to the liver. During IPGTT (Fig. 1a), the HFD-LAu group did not develop glucose intolerance; while the HFD-HAu group had significantly improved glucose clearance at 60–90 min (*P* < 0.05 vs. HFD-C, Fig. 1a). AUC showed similar changes as the blood glucose levels in all three HFD groups (Fig. 1b).

#### Organ distribution of the AuNPs

After 9 weeks, trace amounts of gold were detected in the Chow-C and HFD-C mice (Additional file 1: Table S3) by inductively-coupled plasma-mass spectrometry (Additional file 1), which has also been observed in humans [3]. In both the HFD-LAu and HFD-HAu groups, the highest concentration of gold was found in the abdominal fat tissue, followed by the spleen and the liver (*P* < 0.05 vs. Chow-C and HFD-C, Additional file 1: Table S3). In the HFD-LAu group, gold was negligible in the kidney, brain and heart (Additional file 1: Table S3). In the HFD-HAu group, gold was still detectible in the kidney and brain, but not the heart (*P* < 0.05 vs. Chow-C, HFD-C and HFD-LAu groups, Additional file 1: Table S3).

#### mRNA expression of inflammatory and metabolic markers, and the percentage of macrophages in the fat and liver

**Fig. 2:**
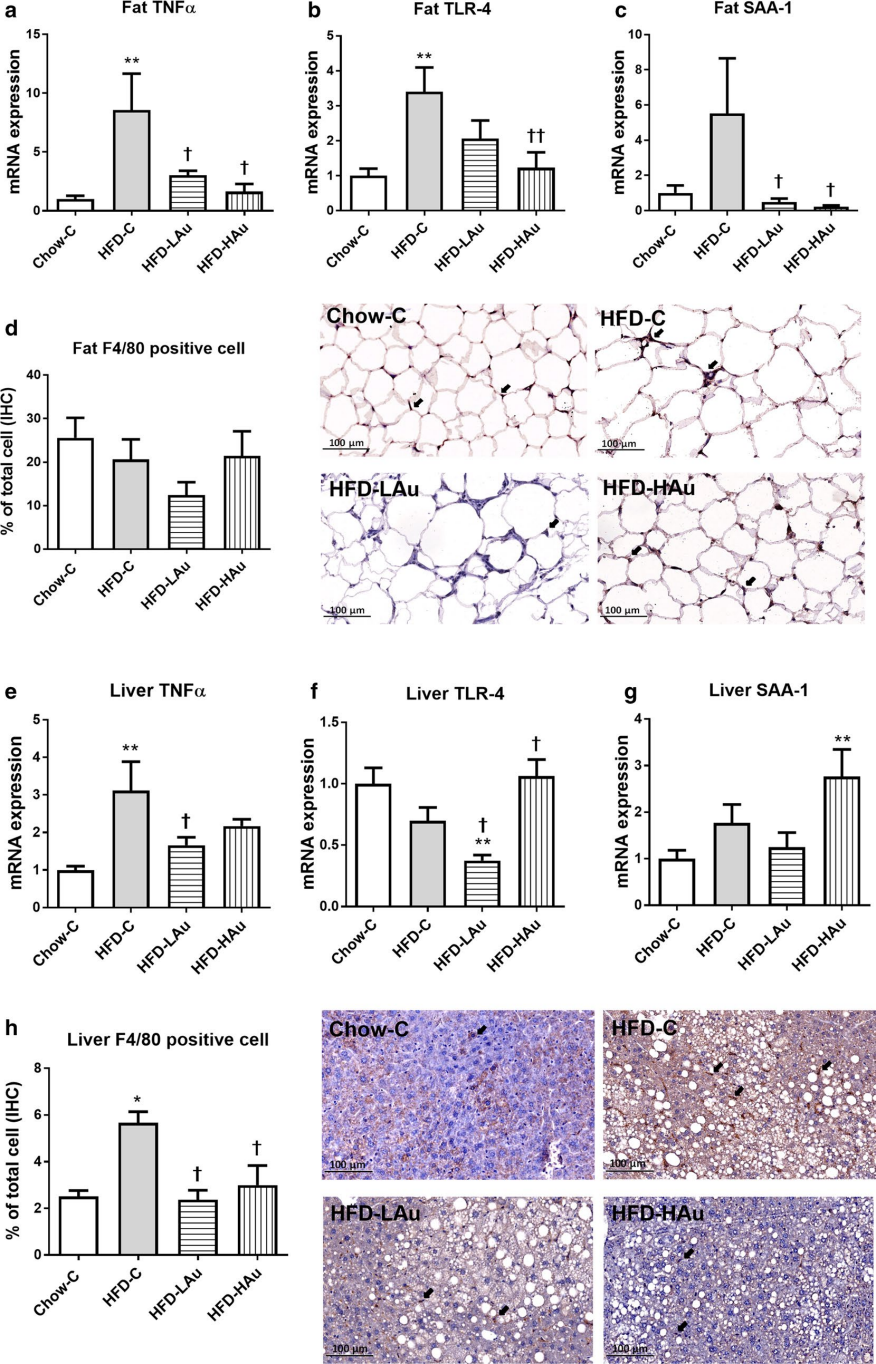
Retroperitoneal fat and liver mRNA expression of (**a**, **e**) TNFα, (**b**, **f**) TLR-4, (**c**, **g**) and SAA-1 in the Chow-C, HFD-C, HFD-LAu, and HFD-HAu mice at 9 weeks of treatment. The percentage of macrophage number and representative image of macrophage number in the abdominal fat (**d**) and liver (**h**) tissues by immunohistochemistry (IHC) staining at the same time point. Results are expressed as mean ± S.E.M, relative to 18 s. Data were analyzed by one-way ANOVA followed by post hoc Bonferroni test. *P < *0.05* vs. Chow-C; **P < *0.01* vs. Chow-C; ^†^*P* < *0.05* vs. HFD-C; ^††^*P* < *0.01* vs. HFD-C. *n* = 5–10

In the retroperitoneal fat, TNFα and TLR-4 mRNA levels were significantly up-regulated following long-term HFD consumption (*P* < 0.05 vs. Chow-C, Fig. 2a, b). On the other hand, serum amyloid A (SAA)-1 level was more than 5 times that of the control mice, however without statistical significance (Fig. 2c). However, the percentage of macrophages was not changed by HFD consumption (Fig. 2d). In the HFD-LAu group, TNFα and SAA-1 mRNA expression levels were significantly down-regulated (*P* < 0.05 vs. HFD-C, Fig. 2a, c); as was TLR-4 level by ~ 50% however without statistical significance (Fig. 2b). In the HFD-HAu group, both TLR-4 and SAA-1 expression levels were significantly reduced (*P* < 0.05 vs. HFD-C, Fig. 2b, c). The percentage of macrophages was halved in HFD-LAu group although without statistical significance, which was not altered in HFD-HAu group (Fig. 2d).

In the liver, HFD consumption alone significantly up-regulated TNFα mRNA expression (*P* < 0.05 vs. Chow-C, Fig. 2e). SAA-1 mRNA levels were nearly doubled in the HFD-C group however without statistical significance (Fig. 2g). The percentage of macrophages was significantly increased by HFD consumption (*P* < 0.05 HFD-C vs. Chow-C, Fig. 2h). Both TNFα and TLR-4 mRNA expression levels were significantly reduced by HFD-LAu treatment; however TLR-4 and SAA-1 expression levels were increased in HFD-HAu group (*P* < 0.05 vs. HFD-C, Fig. 2). AuNP-treatment normalized the percentage of macrophages relative to control animals (*P* < 0.05 vs. HFD-C, Fig. 2h).

**Fig. 3:**
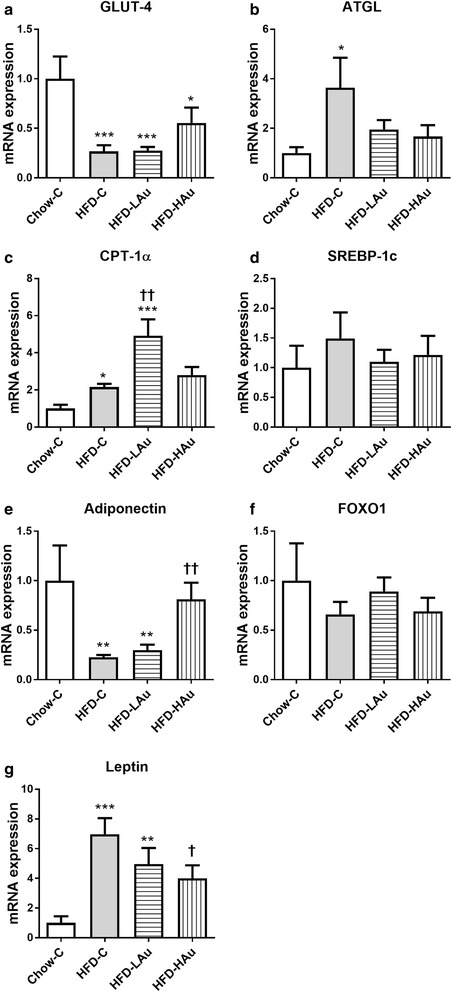
Retroperitoneal fat mRNA expression of **a** GLUT-4, **b** ATGL, **c** CPT-1α, **d** SREBP-1c, **e** adiponectin, **f** FOXO1, and **g** leptin in Chow-C, HFD-C, HFD-LAu, and HFD-HAu mice at 9 weeks of treatment. Results are expressed as mean ± S.E.M, relative to 18 s. Data were analyzed by one-way ANOVA followed by post hoc Bonferroni test. *P < 0.05 vs. Chow-C; **P < 0.01 vs. Chow-C; ^†^*P* < 0.05 vs. HFD-C; ^††^*P* < 0.01 vs. HFD-C; *n* = 5–10

In the fat tissue, mRNA levels of glucose transporter (GLUT)4 and adiponectin were significantly reduced; while ATGL, carnitine palmitoyl transferase (CPT-1α), and leptin were significantly increased following HFD consumption (*P* < 0.05 vs. Chow-C, Fig. 3a–c, g). Conversely, HFD-LAu treatment significantly lowered CPT-1α mRNA expression (*P* < 0.05 vs. HFD-C, Fig. 3c); while HFD-HAu treatment significantly down-regulated leptin, but increased adiponectin mRNA expression (*P* < 0.05 vs. HFD-C, Fig. 3e, g).

**Fig. 4:**
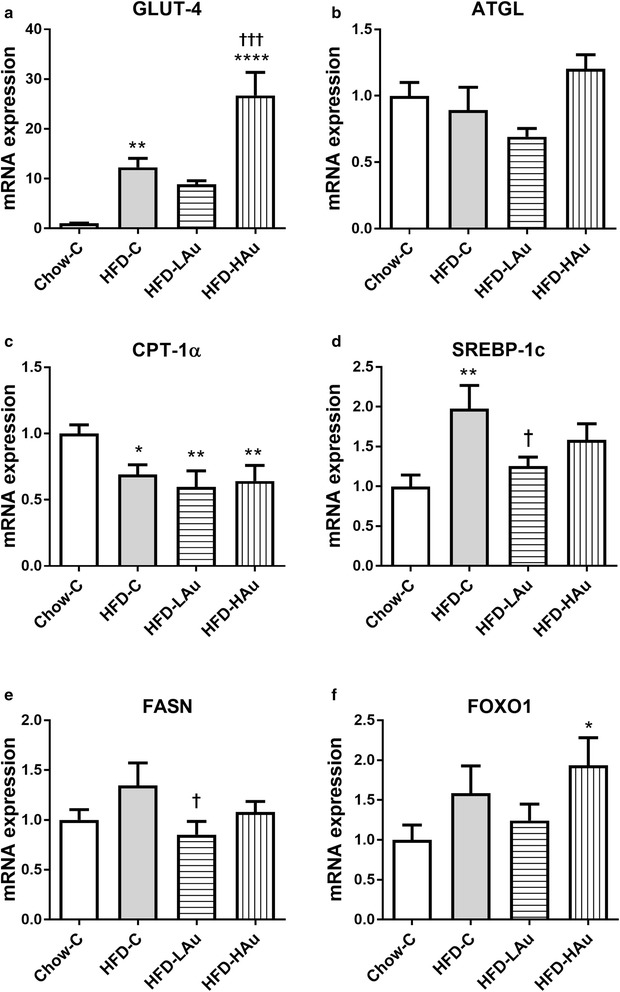
Liver mRNA expression of **a** GLUT-4, **b** ATGL, **c** CPT-1α, **d** SREBP-1c, **e** FASN, and **f** FOXO1 in Chow-C, HFD-C, HFD-LAu, and HFD-HAu mice at 9 weeks of treatment. Results are expressed as mean ± S.E.M, relative to 18 s. Data were analyzed by one-way ANOVA followed by post hoc Bonferroni test. *P < 0.05 vs. Chow-C; **P < 0.01 vs. Chow-C; ^†^*P* < 0.05 vs. HFD-C; ^††^*P* < 0.01 vs. HFD-C; *n* = 5–10

In the liver, GLUT4 and Sterol regulatory element-binding transcription factor (SREBP)-1c mRNA expression levels were significantly up-regulated; while CPT-1α mRNA expression was significantly down-regulated following HFD consumption (*P* < 0.05 vs. Chow-C, Fig. 4a, c, d). Although fatty acid synthase (FASN) was increased by 35% and forkhead box O1 (FOXO1) expression was up-regulated by more than 50%, neither was significant (Fig. 4e, f). HFD-LAu group had significantly reduced SREBP-1c and FASN mRNA expression (*P* < 0.05 vs. HFD-C, Fig. 4d, e). The HFD-HAu group had significantly increased GLUT4 (*P* < 0.05 vs. HFD-C, Fig. 4a), and higher levels of FOXO1 compared to the Chow-C group (*P* < 0.05, Fig. 4f).

### In vitro studies

#### Effects of AuNPs on MΦ cell lines

Low concentration of AuNPs reduced cell viability at 24 and 72 h post incubation (*P* < 0.05 vs. MΦ-C, Additional file 1: Figure S2b, c). Cell viability was reduced in the MΦ-HAu group across all three time points (*P* < 0.05, 0.01 *vs.* MΦ-C, Additional file 1: Figure S2a–c). Reactive oxygen species (ROS) levels were only significantly increased in the MΦ-HAu group at 24 h (*P* < 0.01 *vs.* MΦ-C, Additional file 1: Figure S2e).

TNFα mRNA expression was only significantly reduced in the MΦ-HAu group at 1 h (*P* < 0.05 vs. MΦ-C, Additional file 1: Figure S3a), but was significantly increased in both MΦ-LAu and MΦ-HAu groups at 24 h (*P* < 0.05 vs. MΦ-C, Additional file 1: Figure S3b). TLR-4 mRNA expression was significantly reduced in the MΦ-HAu group at both 1 h and 72 h (*P* < 0.05 vs. MΦ-C, Additional file 1: Figure S3d, f). However, TLR-4 and TNFα protein levels were not changed by AuNPs, which were significantly increased in the positive control LPS incubated cells (*P* < 0.05 vs. MΦ-C, Additional file 1: Figure S4a–c). However, AuNPs cannot suppress LPS induced increase in TLR-4 and TNFα protein levels (data not shown).

#### Effects of AuNPs on 3T3-L1 adipocytes

Cell viability of the mature 3T3-L1 adipocytes (Additional file 1: Figure S5a–c), and 3T3-L1 differentiation from fibroblast (data not shown) were not affected by AuNPs. ROS production was increased in the AD-HAu group at 24 h (*P* < 0.05 vs. AD-C, Additional file 1: Figure S5e). Lipid accumulation was significantly reduced in the AD-HAu group at 1 h (*P* < 0.05 vs. AD-C, Additional file 1: Figure S5g); it was significantly increased by 9% in this group at 72 h (*P* < 0.01 vs. AD-C, Additional file 1: Figure S5i). In addition, adipocyte cell size was increased in the AD-LAu group at 24 h, however it was reduced in the AD-HAu group at 72 h (*P* < 0.05 vs. AD-C, Additional file 1: Table S4). Triglycerides levels secreted into the culture media were similar between the three groups at all time points (Additional file 1: Table S4).

GLUT-4 mRNA levels were significantly down-regulated in both AD-LAu and AD-HAu at 72 h (*P* < 0.01, 0.05 vs. AD-C, Additional file 1: Figure S6c). ATGL was significantly reduced in the AD-LAu group at 24 h (*P* < 0.05 vs. AD-C, Additional file 1: Figure **S**6e). Under low ambient glucose concentration, glucose uptake was significantly reduced in the AD-HAu group at 60 min (*P* < 0.05 vs. AD-C, Additional file 1: Figure S7a); whereas under high ambient glucose concentration, glucose uptake was significantly increased at 5 min in the AD-LAu group (*P* < 0.05 *vs.* AD-C, Additional file 1: Figure S7b).

#### Effects of AuNPs on adipocytes and macrophages in co-culture (MΦ + AD)

In this co-culture system, cell viability and ROS production were similar among the groups at all three time points (Additional file 1: Figure S8). TLR-4 was significantly increased at 24 h in the (MΦ + AD)-HAu group (*P* < 0.05 vs. (MΦ + AD)-C, Additional file 1: Figure S9e). For the metabolic markers, at 24 h GLUT-4 and ATGL mRNA was significantly up-regulated in both (MΦ + AD)-LAu and (MΦ + AD)-HAu groups (*P* < 0.05 vs. (MΦ + AD)-C, Additional file 1: Figure S9b, e). CPT-1α mRNA levels were up-regulated 1.3-fold in the (MΦ + AD)-HAu group versus the control group at 24 h (*P* < 0.05 vs. (MΦ + AD)-C, Additional file 1: Figure S9 h).

## Discussion

In HFD-fed mice, AuNPs slowed down the development of obesity with significantly improved lipid metabolic profile. It also provided a marked protective effect against the development of glucose intolerance, which is recognized as a first step towards type 2 diabetes. In particular, the lower dose provided better outcomes. A reduction in local inflammation within the adipose tissue and the liver may service as the underlying mechanism; while the in vitro co-culturing data support AuNP’s regulation of cellular interactions between macrophages and adipocytes as orchestrating these anti-inflammatory events.

In the current study, the males are not affected by periodical changes in sex hormones and are therefore used for this study to prove the concept. The mice fed a HFD ad libitum for 9 weeks showed a significant increase in their fat mass and developed glucose intolerance, dyslipidemia, and liver steatosis, which are consistent with our previous studies [15, 21, 23]. Liver enzyme levels were also increased by several folds in the HFD-fed mice, suggesting liver cell damage. However, daily AuNP injection significantly ameliorated such effects by HFD consumption, with significant improvement in glucose and lipid metabolism. Liver enzyme changes may suggest a liver protection of AuNPs against dietary lipid influx induced liver damage.

Clinical research suggests that loss of as little as 5% of total body weight can reduce the risk of developing type 2 diabetes by 58% [24]. This benefit was well supported by the current study. The HFD-LAu group showed 8% less body weight and demonstrated normal glucose clearance during IPGTT, while the HFD-HAu group, with 5% less body weight, demonstrated significantly improved glycaemic control. It needs to be noted that this effect was achieved under the condition of free access to HFD without any restriction that employed by the human clinical trial [2]. Their daily caloric intake was even higher than non-treated mice consuming HFD. This may be an adaptation to their reduced fat mass; where smaller fat mass may be due to increased CPT-1α expression to increase fatty acid oxidation for energy synthesis. Therefore, it can be postulated that combining the AuNP treatment with restricted energy intake to the level of the Chow-C group may exert more pronounced weight loss effect. This is yet to be confirmed by future studies. The low concentration of AuNP seems to exert a better effect than the high concentration. This may be due to the aggregative nature of the AuNPs at high concentration, which results in less free monodispersed AuNPs entering the tissue and the circulation, as well as impacting on the cells. The effects of AuNP are well known to be highly dependent on particle size [25]. As this was the first study to show the anti-obesity effect of the AuNPs, ip injection was chosen as it is the most convenient method of AuNP delivery. In future studies, we will test the efficacy of subcutaneous injection and oral delivery, which are the common administration methods in humans. In addition, for unknown reasons, the lower dose AuNPs seems to stimulate insulin secretion, which may have contributed to normalized glycaemic control in this group. This result warrants further investigation of the interaction between AuNPs and β-cells.

Increased macrophage infiltration has been suggested to contribute to the low-grade inflammation state commonly associated with obesity [26]. During HFD consumption, excessive fat accumulation in the abdominal fat tissue increases the recruitment of ATMs [27], producing pro-inflammatory cytokines (e.g. TNFα), which in turn drives obesity-related metabolic disorders [27–31]. TNFα is known to reduce free fatty acid transporter and extracellular lipoprotein lipase activity, thereafter inhibit the uptake of fatty acids into fat cells, leading to hyperlipidemia and ectopic lipid storage (e.g. in the liver); while local lipid accumulation is a key contributor to insulin resistance [32]. TNFα itself can also interrupt insulin signaling, causing reduced glucose uptake [33]. In this study, F4/80 expressing macrophages were increased in the liver following HFD consumption, and this was reduced by the treatment with AuNPs, demonstrating a direct anti-inflammatory effect. The percentage of F4/80 positive macrophages were not increased by HFD consumption in the abdominal fat tissue. Longer HFD feeding duration may be need to observe increased macrophages in the fat tissue as shown in the other study, while the macrophages are not the only immune cells in the fat causing inflammatory responses [34]. We think that the increase in the liver and not in the fat represents different recruitment dynamics in this model. Irrespective of macrophage accumulation, TNFα and upstream TLR-4 mRNA expression were both increased, which may reflect increased M1 macrophage activity. As such, fat derived adiponectin (insulin sensing promotor) and GLUT4 (insulin dependent glucose transporter) were significantly down-regulated in the HFD-C mice, resulting in glucose intolerance. The up-regulation of ATGL, CPT-1α and leptin in the fat tissue reflects an increase in lipid influx into the adipocytes, while increased ATGL may contribute to nearly doubled blood NEFA levels following HFD consumption. Similar changes in TNFα were seen in the liver, resulting from excessive liver lipid storage which would activate the Kupffer cells (liver macrophage-like cells) [35]. This inflammatory response in turn stimulates SREBP-1c which further activates FASN activity to increase lipogenesis [36], leading to a fatty liver [36, 37]. Regardless of the functionality of the nanoparticles, it has been suspected 99% of the nanoparticles administered in vivo will be uptaken by the macrophages [38]. This study strongly points to an anti-inflammatory effect upon the macrophage cells by the AuNPs, via suppressing pro-inflammatory cytokine production in both the fat and liver tissues, regardless of the impact on macrophage numbers.

Interestingly, the changes in metabolic markers were not consistent in the HFD-LAu and HFD-HAu groups, suggesting different working mechanisms. In the HFD-Lau group, increased fat CPT-1α may increase lipid oxidation, resulting in a better blood lipid profile and smaller fat mass [39]. Upon AuNP treatment, liver lipogenesis appeared to be suppressed with a synchronized down-regulation of SREBP-1c and FASN mRNA levels. Based on these observations, we propose that low dose AuNP could reduce hepatic ectopic lipid deposition to impede the development of obesity-associated fatty liver disease. In the HFD-HAu group, increases in fat GLUT4 and adiponectin is suggestive of an improved insulin response and glucose uptake. There was a drastic increase in GLUT4 by AuNP treatment in this group, which may contribute to significantly improved glucose clearance during IPGTT.

The in vitro study allowed us to examine the impact of AuNPs on individual cell types, as well as their interactions via the use of a contact co-culture system. Interestingly, AuNPs induced inflammatory responses in macrophages cultured alone as foreign objectives; however, this response seemed to be suppressed when grown in the presence of adipocytes. Increased oxidative stress has been suggested to be the major cause of organ toxicity [40]. Increased ROS production appeared in macrophages treated with high concentration of AuNP in line with reduced cell viability, consistent with the literature [41, 42]; however such changes diminished with the co-culture with adipocytes suggesting unknown antioxidative mechanism due to the interaction between these two cell types. Similarly, AuNP treatment of adipocytes cultured alone did not change their differentiation rate into mature adipocytes, nor metabolic markers. However, it did result in reduced lipid droplet size, which may contribute to slow-down fat accumulation during HFD consumption. On the other hand, AuNP treatment of adipocytes co-cultured with macrophages resulted in metabolic marker change that may potentially improve lipid metabolism as well as glucose uptake. Given that the co-cultured adipocyte and macrophage more closely resembles conditions in vivo, this suggests that the same interactions may be occurring within the mice treated with AuNPs. These studies also highlight the limitation of using single-cell culture systems. Additionally, these changes were more prominent at 24 h, suggesting daily administration of the AuNPs is desired to exert a continuous and more refined metabolic effect.

Neutralization of circulating TNFα alone has been shown to increase insulin sensitivity and glucose uptake in peripheral tissues, although to date, such approaches have not been successfully translated into humans [27, 30, 31]. This is perhaps due to the involvement of other pro-inflammatory cytokines yet to be defined. Therefore, altering macrophage responses or phenotype may be the key to inhibit systemic inflammatory processes. AuNPs emerge as highly suitable candidates to carry out this task, with both TNFα and TLR-4 down-regulated upon AuNP administration, consistent with our previous acute study in lean mice [6]. In the study by Kosteli et al. [9], at early stage of weight loss, increased lipolysis can recruit both macrophages and T cells. However, different from weight gain, most of these adipose macrophages during weight loss are not pro-inflammatory as shown by reduced TNFα expression in the fat, which functions as lipid scavenger during increased lipolysis due to weight loss. In Kosteli’s paper [9], the depletion of macrophages in the fat tissue has been shown to prevent weight loss, suggesting the important role of M2 type macrophages in initiating fat loss. In our study, we also observed significantly suppressed TNFα expression by AuNP treatment, although macrophage numbers were not markedly reduced. This suggests that after engulfing the AuNPs, adipose macrophages may be switched from M1 to M2 phenotype to facilitate fat loss, or the function of M2 macrophages is improved by the AuNPs to suppress inflammation. Indeed, another study has shown that AuNPs are preferably taken up by M2 than M1 macrophages [43]. However, to prove such a hypothesis still requires further comprehensive studies, which are beyond the scope of this study. Future studies can remove the limitation imposed by use of only one size and shape AuNP in this study. Such future work could determine whether other types of nanoparticles can exert a similar metabolic benefit.

The uptake and elimination of the gold from tissues is still a key issue when considering long-term treatments. In line with previous studies, AuNPs were taken up into the surrounding abdominal fat after repeated IP administration, which were then able to enter the blood stream, from which they then distribute and accumulate within other organs [6, 44].

## Conclusions

In conclusion, the alterations in the local pro-inflammatory cytokine environment by AuNPs may be the key underlying mechanism for the weight reduction in HFD-fed mice. Specifically, AuNP-treated mice were protected against the development of HFD-induced glucose intolerance as well as hyperlipidemia. AuNPs may serve as a new paradigm to inspire treatments for weight loss and the prevention of obesity-related metabolic disorders and as a useful research tool to probe biological mechanisms.

## Additional file

**Additional file 1.** Additional material.
