# Supplementary material for: Gold nanoparticles improve metabolic profile of mice fed a high-fat diet
Source: J Nanobiotechnology. 2018 Feb 6;16:11. doi: 10.1186/s12951-018-0338-1 (PMC5800098; doi:10.1186/s12951-018-0338-1)
Supplement: Supplementary file 1 — Additional file 1. Additional material. [file 12951_2018_338_MOESM1_ESM.pdf]

Supplementary materials

Gold-nanoparticles improve metabolic profiles in high-fat diet mice

Hui Chen [1,2*], Jane P M Ng [1*], Yi Tan [1], Kristine McGrath [1,2], Brian Oliver[1,2], David P Bishop [3], Michael B Cortie [4], Bruce K Milthorpe [1,2], Stella M Valenzuela [1,2]

1. Molecular Biosciences Team, School of Life Sciences, Faculty of Science, University of Technology Sydney. NSW 2007, Australia
2. Centre for Health Technology, University of Technology Sydney, NSW 2007, Australia
3. School of Mathematical and Physical Sciences, Faculty of Science, University of Technology Sydney, NSW, 2007, Australia
4. Institute for Nanoscale Technology, University of Technology Sydney. NSW 2007, Australia

[*] *These authors contributed equally to this work.*

Corresponding Author

Associate Professor Stella M Valenzuela, Molecular Biosciences Team, School of Life Sciences, Faculty of Science, University of Technology Sydney. NSW 2007, Australia. Phone: 61 2 9514 1917. Fax: 61 2 9514 8206. E-mail: Stella.Valenzuela@uts.edu.au

Running title: Gold-nanoparticles in treating obesity

Supplementary Materials and Methods

Histology analysis:

Fixed abdominal fat tissue samples were embedded in paraffin and sectioned. Adipocyte cell size was measured in hematoxylin and eosin (H&E) stained tissues by quantification of >800 cells from each section by Image J software (Image J, National Institutes of Health, MD, USA).

In vitro experiments:

The RAW264.7 macrophage cells (MΦ, originate CL-173, ATCC®, Manassas, VA, USA) were kindly given by A/Prof O'Brien (University of Technology Sydney). The Murine 3T3-L1 fibroblast cells (adherent, orginate TIB-71, ATCC®, Manassas, VA, USA) were kindly given by Dr. Fei (University of New South Wales). All cell-lines were maintained at 5% CO_2 and 37°C. Cell viability was determined using 3-(4,5-dimethylthiazol-2-yl)-2,5-diphenyltetrazolium bromide (MTT) assay (Sigma Aldrich, St. Louis, MO). MΦ cell line was maintained in DMEM supplemented with fetal bovine serum andpenicillin/streptomycin. MΦ were treated with low dose (MΦ-LAu, 0.315μg/ml) and high dose (MΦ-HAu, 3.15μg/ml) of AuNPs. PBS treatment was used as negative control (MΦ-C). Lipopolysaccharide (LPS) treatments (1ng/ml and 10μg/ml) were used as positive controls. The cells were harvested at 1, 24, and 72 h.

Murine 3T3-L1 fibroblast was differentiated into mature adipocytes using a previously published protocol [1,2]. The differentiated mature 3T3-L1 adipocytes were treated with the same two doses of AuNPs as the MΦ, yielding three groups: control (AD-C); low dose AuNP (AD-LAu, 0.315μg/ml); high dose AuNP (AD-HAu, 3.15μg/ml) and collected at the same time points. In another experiment, pre-adipocytes were continuously treated with AuNPs (3.15μg/ml, every second day) during the differentiation from day 0. The cells were collected when the adipocytes matured at day 8.

MΦ and mature 3T3-L1 adipocytes were co-cultured with a starting ratio of 1:15 as per published protocol [3]. The macrophage and adipocyte co-culture system was treated with the same two doses of AuNPs as stated above.

Inductively-coupled plasma-mass spectrometry (ICP-MS): Tissue samples (~0.02–0.1 g) were dehydrated using a series of graded alcohols and freeze drying (Alpha 1-2 LDplus freeze dryer, CHRIST® GmbH, Osterode am Harz, Germany). Dried tissues were powdered and step digested in a highly corrosive mixture of nitric acid (HNO_3), hydrochloric acid (HCl), and hydrogen peroxide (1:2:1, v:v:v) from Baseline® Seastar Chemicals, BC, Canada. The acid-digested samples were then

diluted with equal volume of water and refrigerated for subsequent analysis using ICP-MS as per previous publication[4]. Agilent Technologies 7500cx ICP-MS (Agilent Technologies, VIC, Australia) was used with sample introduction via a MicroMist concentric nebulizer (Glass Expansion, VIC, Australia) and a Scott type double pass spray chamber cooled to +2°C. The ICP-MS extraction lens conditions were selected to maximize the sensitivity of a 1% HNO_3/HCl (1:2, v:v) (Choice Analytical, NSW, Australia). Helium was added into the octopole reaction cell to reduce interference. Calibration curves were constructed using a certified Au calibration standard (Choice Analytical, NSW, Australia) matrix matched with a concentration range (0–1,000 ng mL^{-1}). The results were analyzed using Agilent Technologies MassHunter Workstation software.

Cellular oxidative stress: The concentration of intracellular ROS was determined using a modified 2',7'-dicholorofluorescein diacetate (H_2-DCFDA, Sigma Aldrich, MO, USA) method. Briefly, all the cells were incubated with H_2-DCFDA (10 μM) in DPBS for 12 min in the dark. The fluorescence signal was measured (485 nm excitation, 530 nm emission) using Infinite M200® Pro plate reader (Tecan Deutschland GmbH, Crailsheim, Germany). ROS was adjusted to the cell viability of adjacent wells and expressed as fold differences relative to the control.

Western blot analysis: Whole cell protein lysate was extracted from RAW264.7 macrophages using RIPA lysis buffer (50 mL) and protease inhibitor cocktail (Sigma Aldrich, MO, USA). Protein samples were separated on NuPage® Novex® 4-12% Bis-Tris gels (Life Technologies, CA, USA) and then transferred to PVDF membranes (Thermo Scientific, MA, USA), which were then incubated with the primary antibodies (TNFα (1:1,000, Merck Millipore, MA, USA); TLR-4 (1:500) and β-actin (1:1,000, Santa Cruz Biotechnology, TX, USA)), followed by secondary antibodies (goat anti-rabbit or goat anti-mouse IgG horseradish peroxidase-conjugated secondary antibody (1:5,000–10,000, Santa Cruz Biotechnology, TX, USA).

Cellular glucose uptake: Glucose uptake was measured in mature adipocytes pre-treated with PBS

(AD-C), low dose of AuNP (AD-LAu, 0.315 μg mL^{-1}) or high dose of AuNP (AD-HAu, 3.15 μg mL^{-1}) for 24 h. The adipocytes were then incubated at difference ambient glucose concentrations (5 mM and 10 mM) and insulin (100 nM) for 5, 20, and 60 min.

Supplementary Results

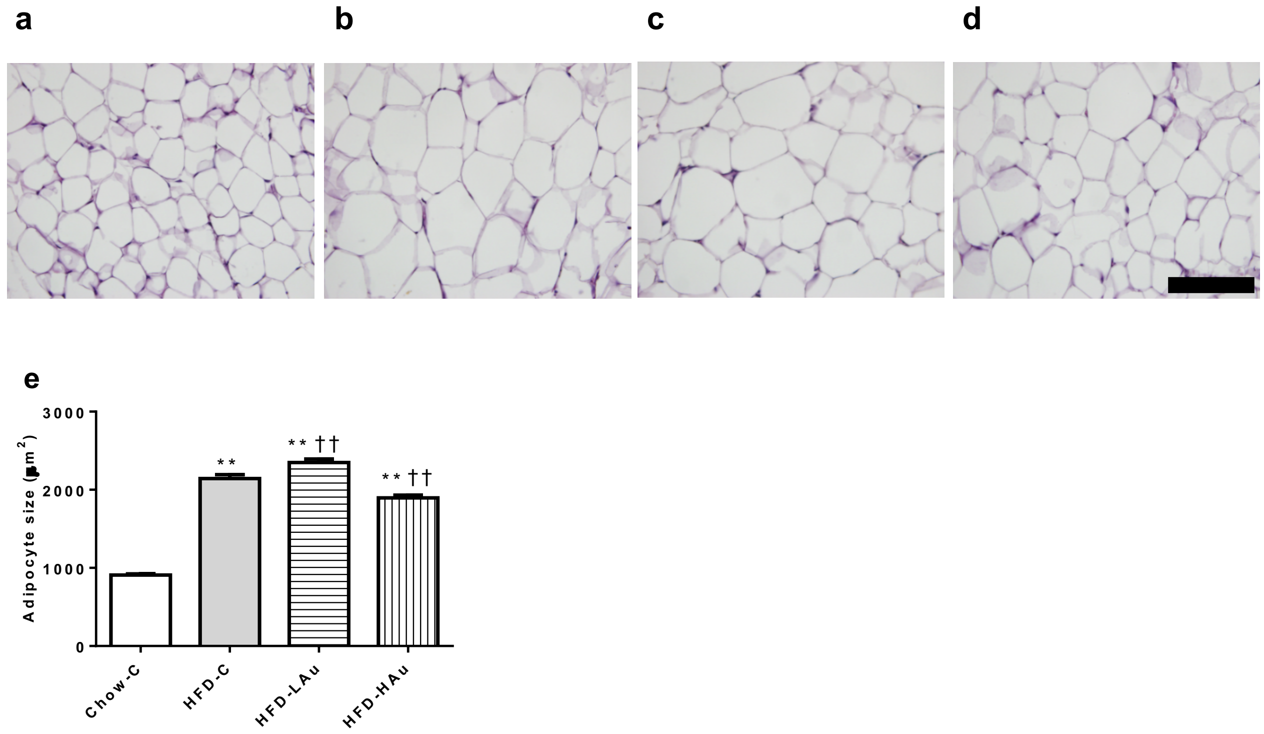

Supplementary Figure S1 Effects of 9 weeks HFD and AuNP treatment on the size of mesenteric adipose tissue determined by H&E. Representative image of mesenteric adipose tissue stained with H&E for (a) Chow-C, (b) HFD-C, (c) HFD-LAu and, (d) HFD-HAu mice at 9 weeks. Images were taken at 40x magnification, scale bar=100 μm for (a–d). (e) Adipocyte size measured from H&E tissue sections by ImageJ. Results are expressed as mean ± S.E.M. Data were analysed by one-way ANOVA followed by *post hoc* Bonferroni test, ** *P<0.01 vs.* Chow-C; †† *P<0.01 vs.* HFD-C; *n*=3.

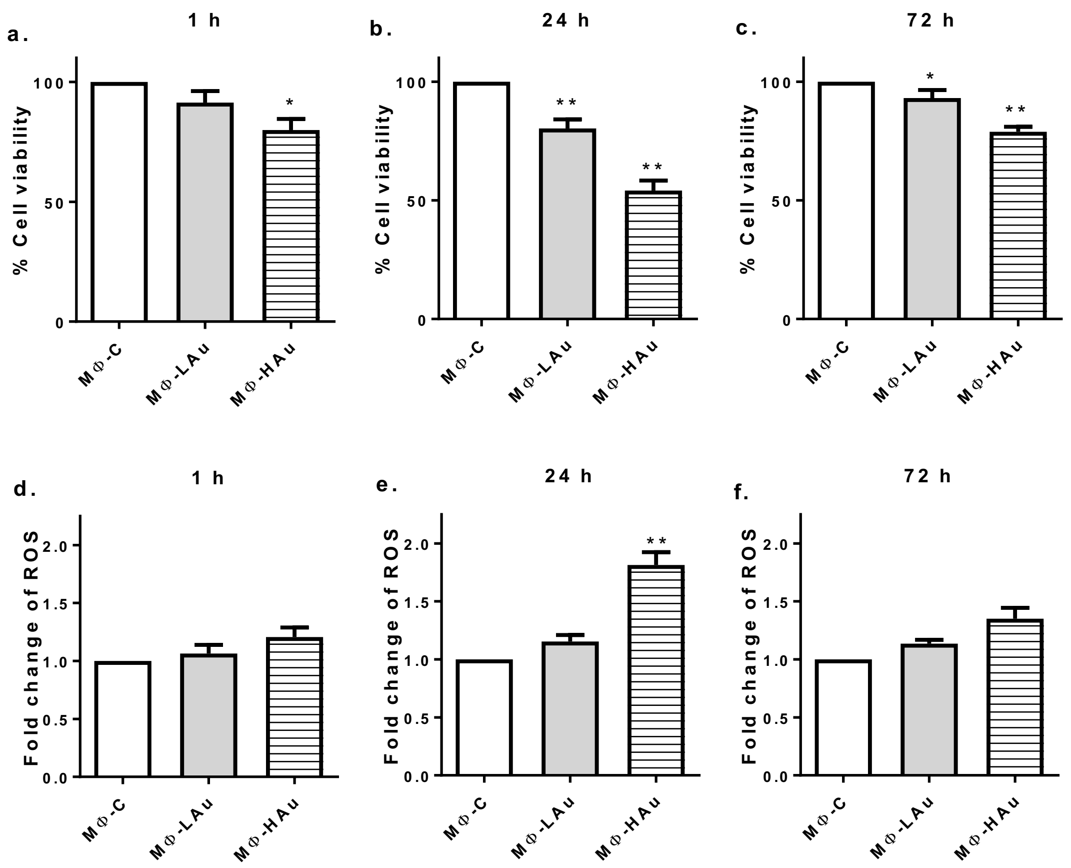

Supplementary Figure S2: Cell viability (a–c) and fold changes in ROS production standardized by cell viability (d–f) in AuNP-treated RAW 264.7 macrophages (MΦ) in at 1, 24 and 72h. Results are expressed as mean ± S.E.M. Data were analyzed by one-way ANOVA followed by *post hoc* Bonferroni test, * $P<0.05$ *vs.* MΦ-C; ** $P<0.01$ *vs.* MΦ-C; n=3–4. MΦ-C: control; MΦ-LAu: 0.315 µg/ml, low dose AuNP treatment; MΦ-HAu: 3.15 µg/ml, high dose AuNP treatment.

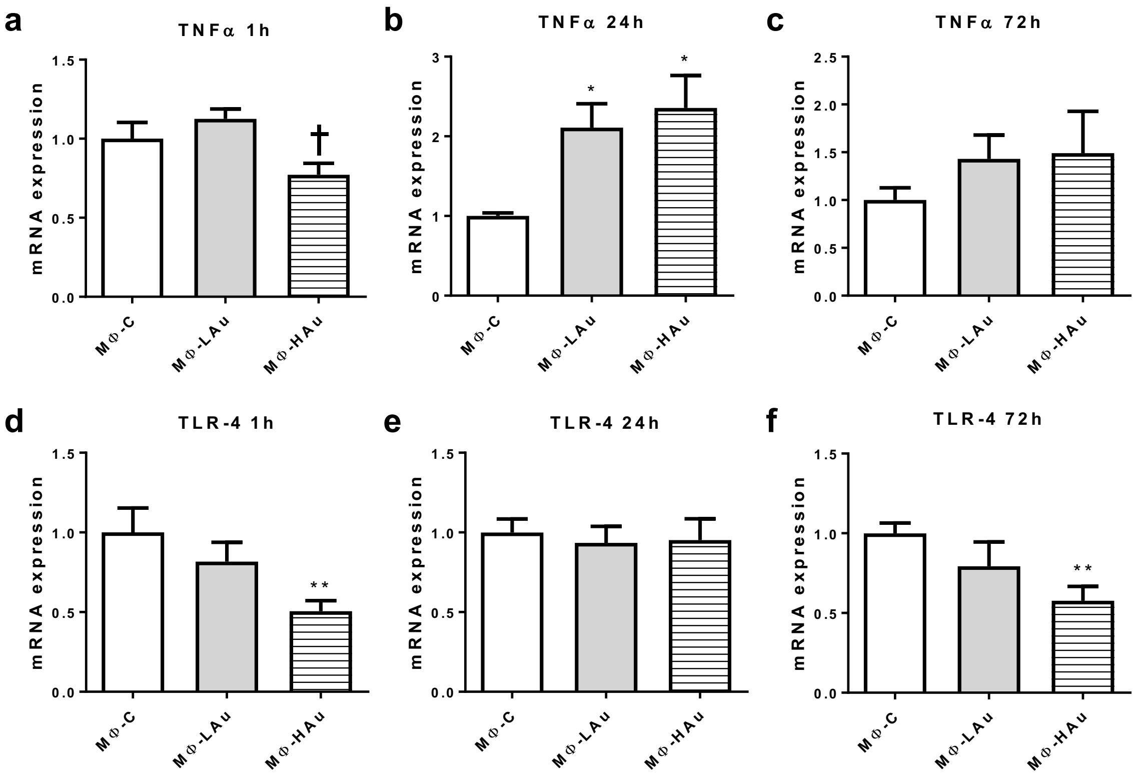

Supplementary Figure 3S: mRNA expression of (a-c) TNFα and (d-f) TLR-4 in AuNP-treated RAW 264.7 macrophages control (MΦ-C), low dose AuNP (MΦ-LAu, 0.315 µg/ml, and high dose AuNP (MΦ-HAu, 3.15 µg/ml) cultures at 1, 24 and 72h. Results are expressed as mean ± S.E.M, relative to 18s. Data were analyzed by one-way ANOVA followed by *post hoc* Bonferroni test.

* *P<0.05 vs.* MΦ-C; ** *P<0.01 vs.* MΦ-C, † *P<0.05 vs.* MΦ-LAu; *n*=10.

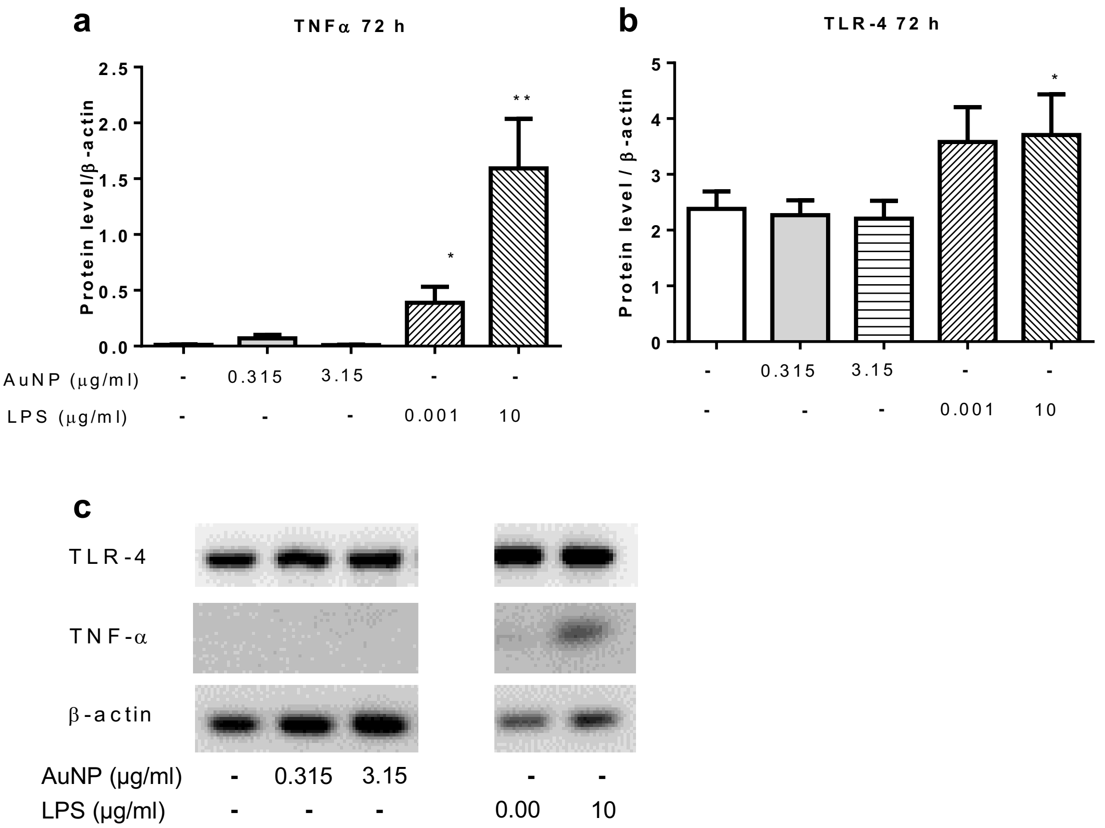

Supplementary Figure S4: Effect of AuNP treatment on TLR-4 (a) and TNFα (b) protein levels in RAW 264.7 macrophages (MΦ). (**c**) shows the representative western blots. Results are expressed as mean ± S.E.M. Data were analyzed by one-way ANOVA followed by *post hoc* Bonferroni test, * *P<0.05 vs.* control; ** *P<0.01 vs.* control; *n*=4. MΦ-C: control; MΦ-L: 0.315 μg/ml, low dose AuNP treatment; MΦ-H: 3.15 μg/ml high dose AuNP treatment.

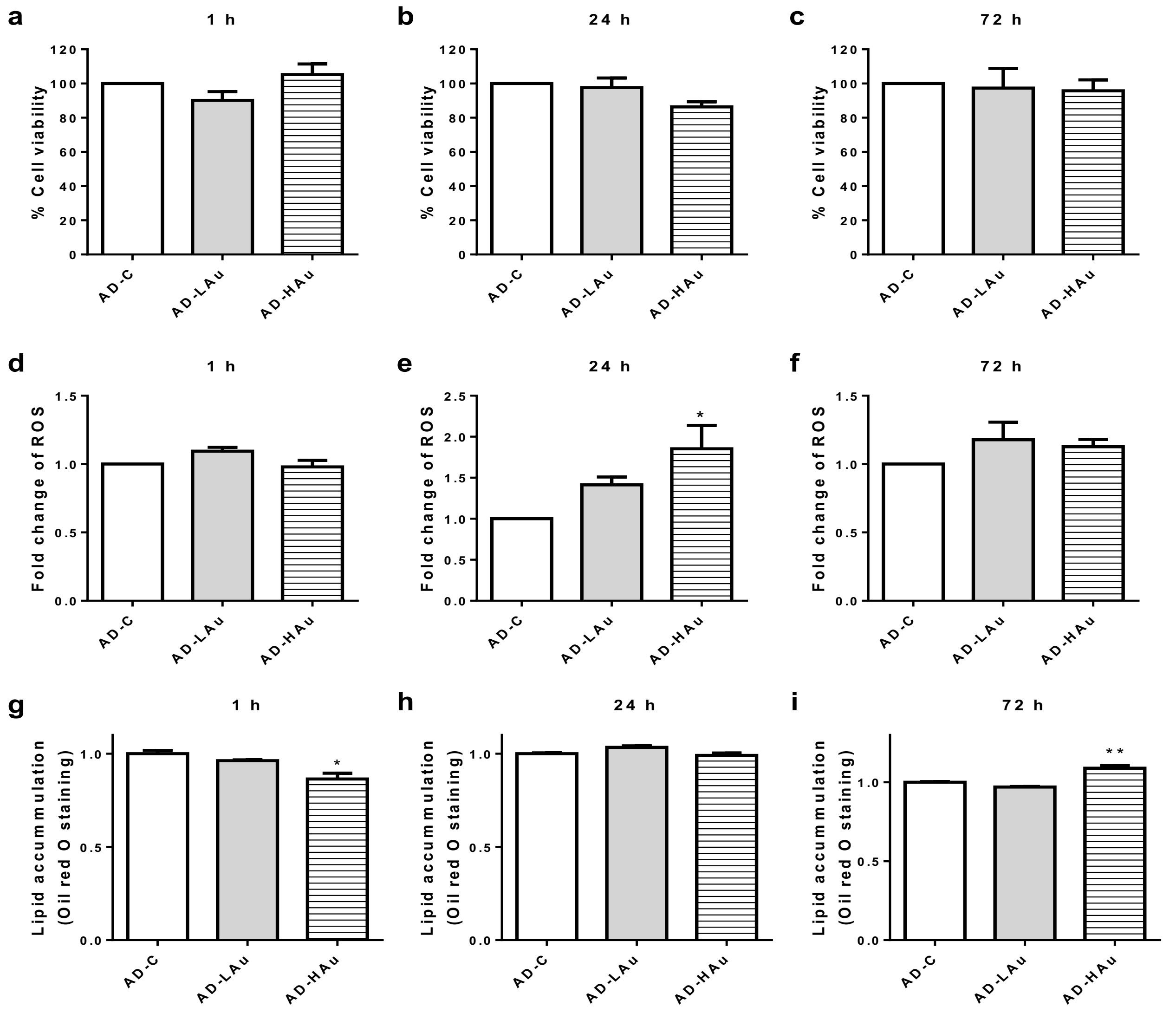

Supplementary Figure S5: Cell viability (a–c), ROS productions standardized by cell viability (d–f), and lipid accumulation (g–i) in mature 3T3-L1 adipocytes control (AD-C), 0.315 µg/ml AuNP (AD-LAu) and 3.15 µg/ml AuNP (AD-HAu) cultures at 1, 24 and 72 h. Results are expressed as percentage mean ± S.E.M. Data were analyzed by one-way ANOVA followed by *post hoc* Bonferroni test, * *P<0.05 vs.* AD-C; ** *P<0.05 vs.* AD-C; *n*=2–3.

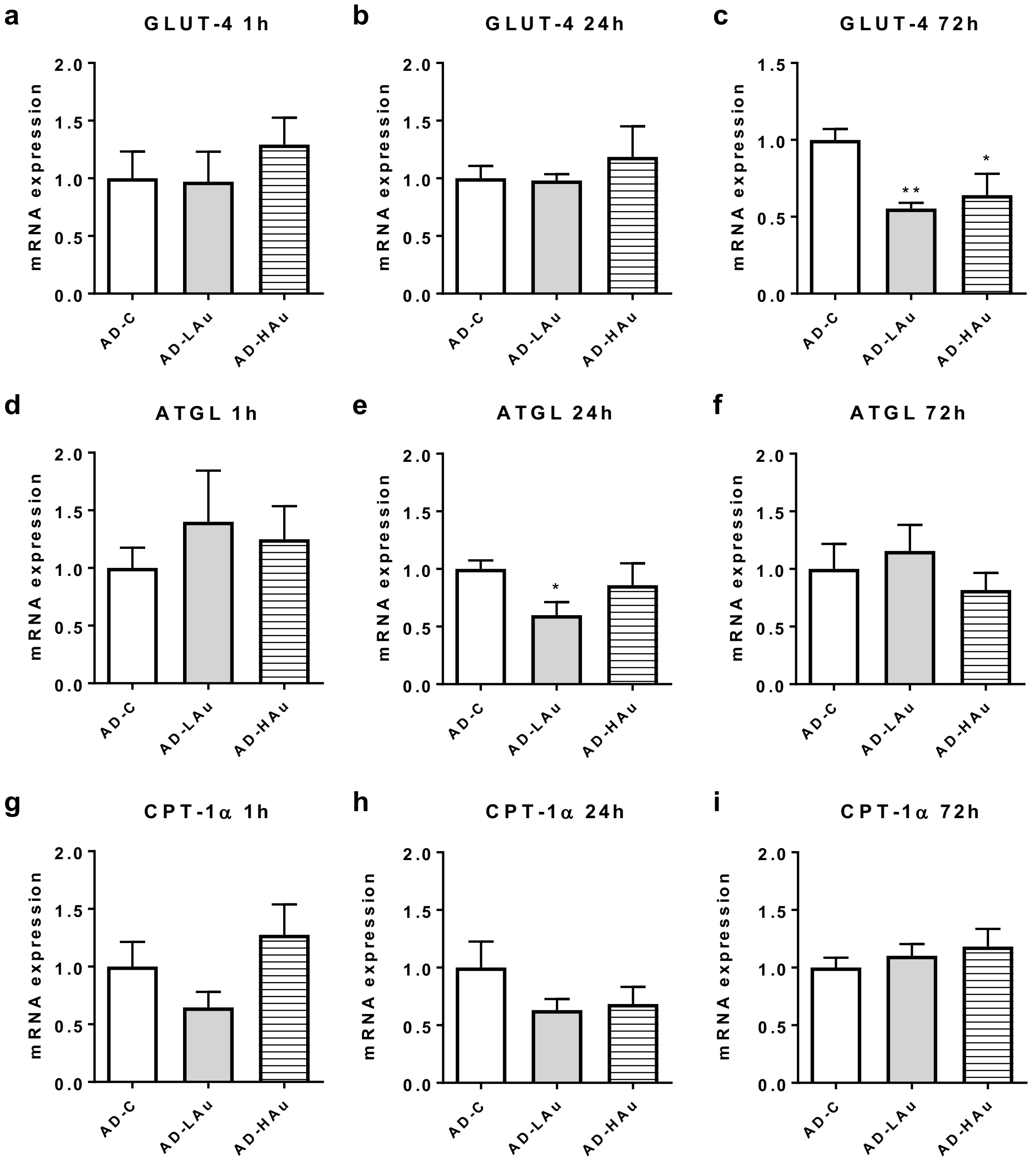

Supplementary Figure S6: mRNA expression of (a-c) GLUT-4, (d-f) ATGL, and (g-i) CPT-1α in 3T3-L1 adipocytes with control (AD-C), low dose AuNP (AD-LAu, 0.315 µg/ml), high dose AuNP (AD-HAu, 3.15 µg/ml) treatment at 1, 24 and 72h. Results are expressed as mean ± S.E.M, relative to 18S. Data were analyzed by one-way ANOVA followed by *post hoc* Bonferroni test. * *P<0.05 vs.* AD-C, ** *P<0.01 vs.* AD-C; *n*=8.

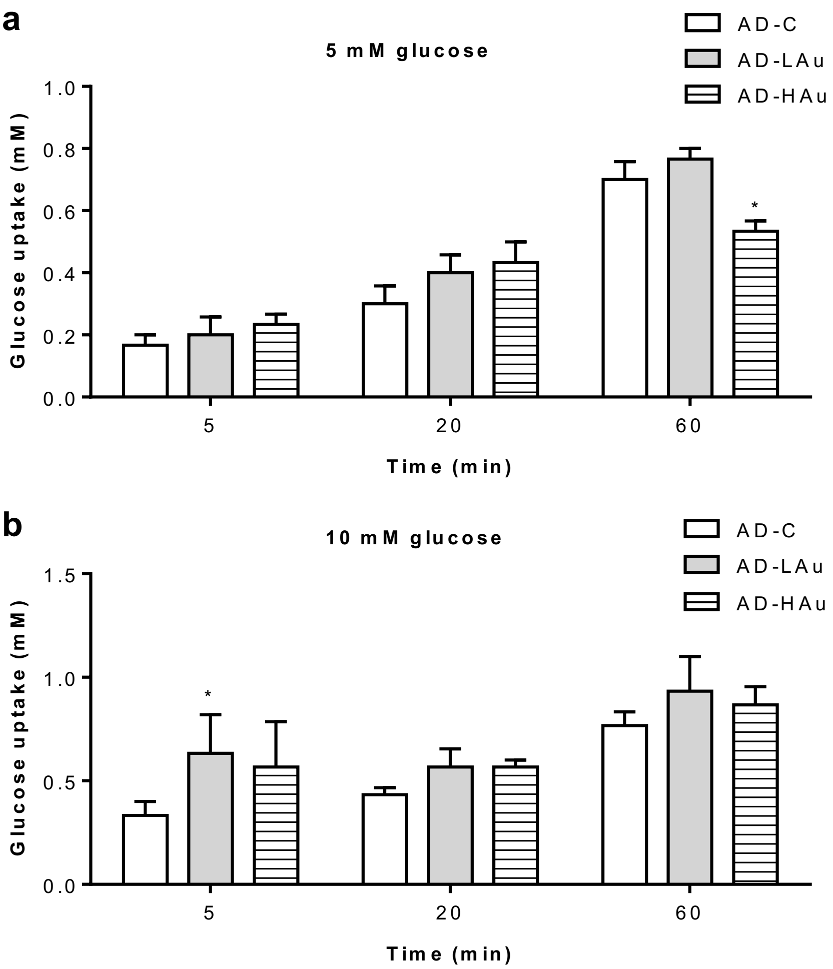

Supplementary Figure S7: Insulin-mediated glucose uptake in mature adipocytes pre-treated with PBS (AD-C), 0.315 µg/ml AuNP (AD-LAu), and 3.15 µg/ml AuNP (AD-HAu) for 5, 20, and 60min, in ambient glucose concentrations of 5mM (a) and 10mM (b). Results are expressed as mean ± S.E.M. Data were analyzed by one-way ANOVA with repeated measures followed by *post hoc* Bonferroni test at each time point, * $P<0.05$ *vs.* AD-C; n=3.

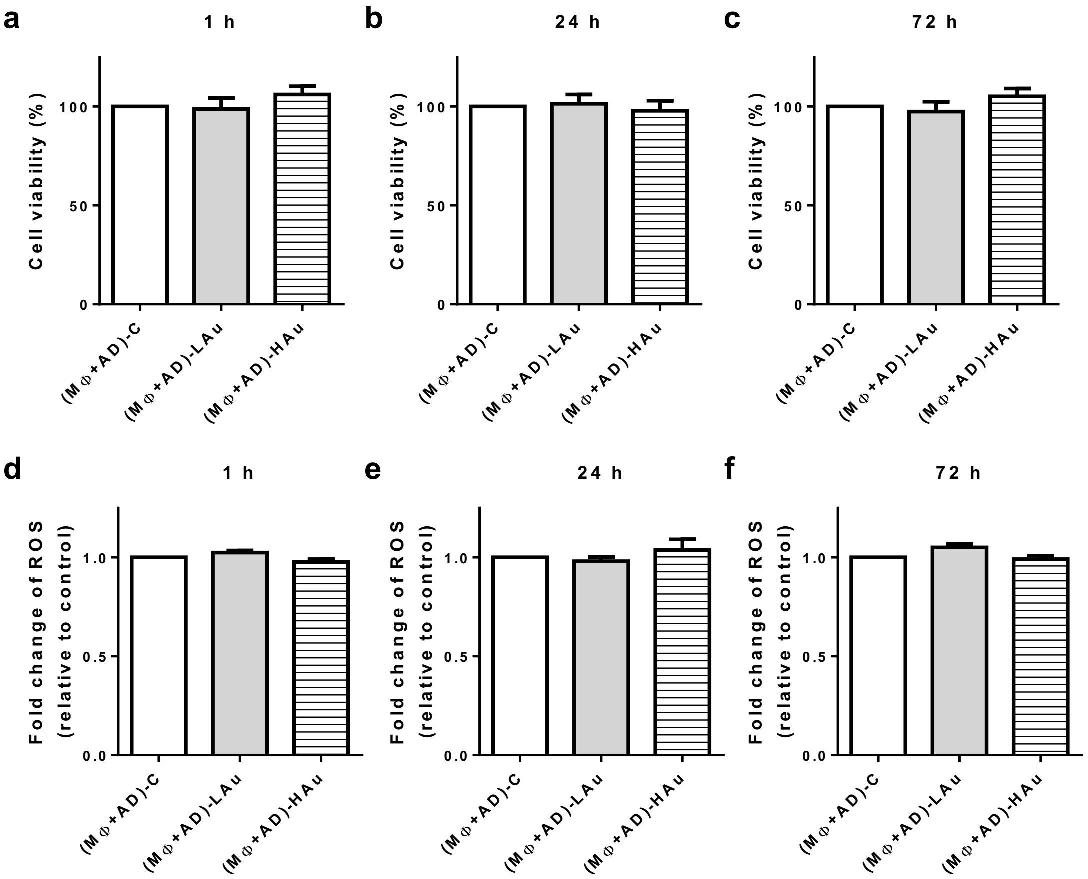

Supplementary Figure S8. Cell viability (a–c) and ROS productions standardized by cell viability (d–f) in AuNP treated 3T3-L1 adipocytes and RAW 264.7 macrophages co-culture control (MΦ+AD)-C, 0.315 µg/ml AuNP (MΦ+AD)-LAu, and 3.15 µg/ml AuNP (MΦ+AD)-HAu at 1, 24h, and 72h. Results are expressed as mean ± S.E.M. Data were analyzed by one-way ANOVA followed by *post hoc* Bonferroni test; * $P<0.05$ *vs.* AD-C; $n=3$.

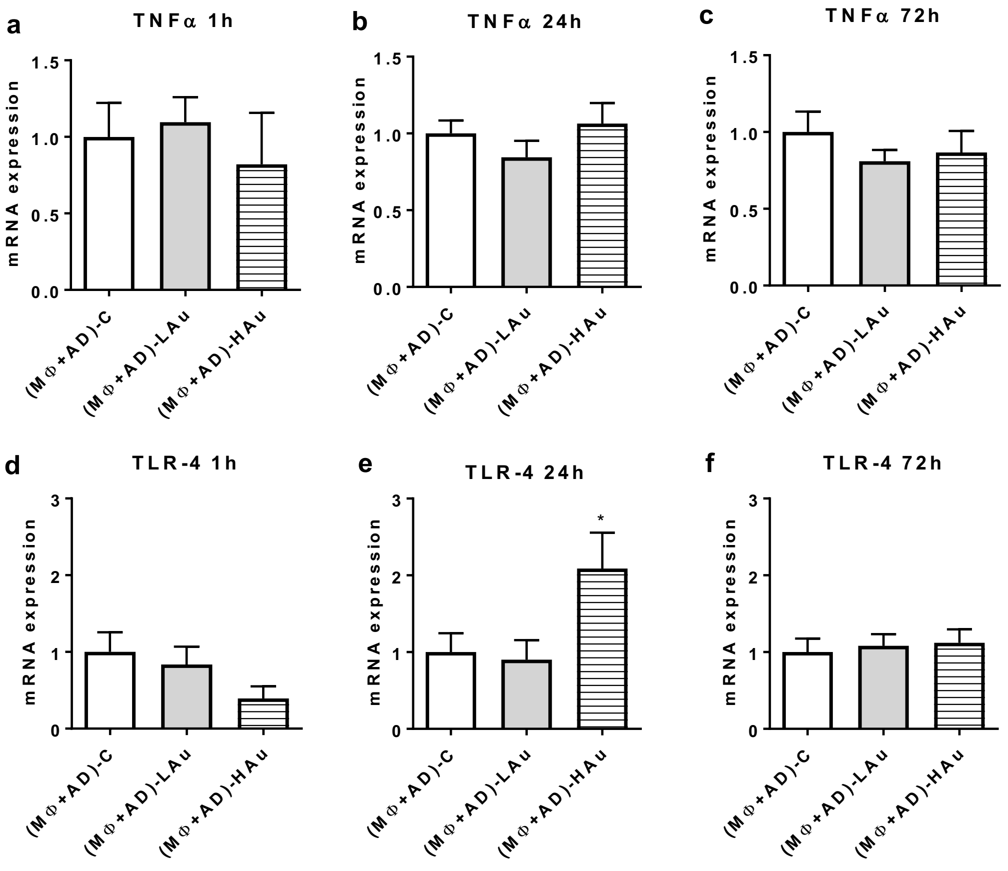

Supplementary Figure S9: mRNA expression of pro-inflammatory markers in AuNP-treated 3T3-L1 adipocytes and RAW 264.7 macrophages co-culture (MΦ+AD) at 1h (a,d), 24h (b,e), and 72h (c,f). Results are expressed as mean ± S.E.M, relative to 18s. Data were analyzed by one-way ANOVA followed by *post hoc* Bonferroni test, * $P<0.05$ *vs.* (MΦ+ AD)-C; n=10/group.

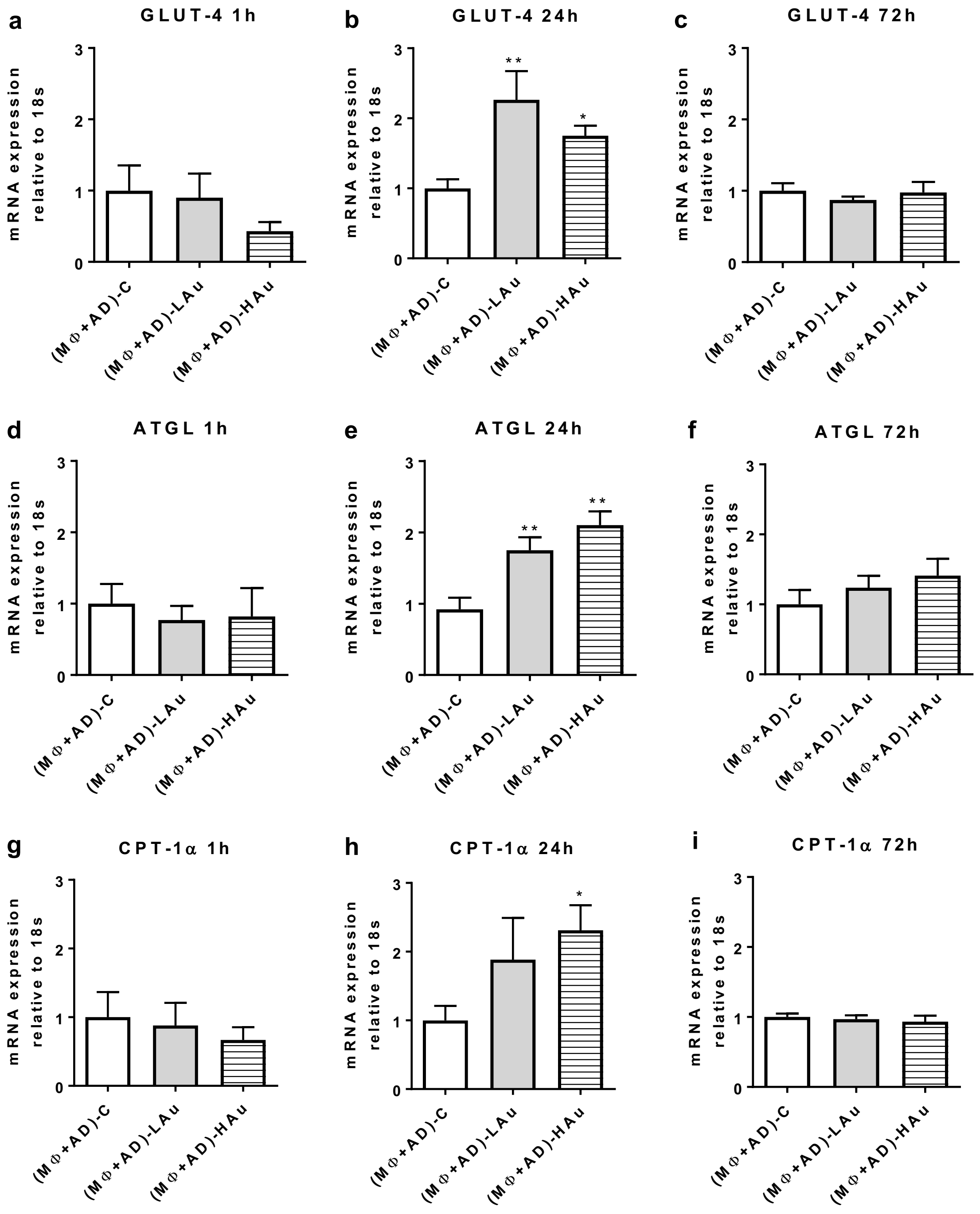

Supplementary Figure S10 mRNA expression of adipokines in AuNP-treated 3T3-L1 adipocytes and RAW 264.7 macrophages co-culture (MΦ+AD) at 1h (A–D), 24h (E–H), and 72h (I–L). Results are expressed as mean ± S.E.M, relative to 18s. Data were analyzed by one-way ANOVA followed by *post hoc* Bonferroni test, * *P<0.05 vs.* (MΦ+AD)-C; *n*=10.

Synthesis of gold nanoparticles:

A quantity of colloidal suspension of gold nanoparticles was prepared by repeat applications of the 'citrate' method. Briefly, glassware, thermometer and Teflon stirrer bar were cleaned in *aqua regia* (3 parts of concentrated HCl plus to one part of concentrated HNO_3) before use. (Extreme cleanliness is essential if a stable sol is required.) Tetrachloroauric acid ($HAuCl_4.3H_2O$) was used to make up a 1 mM starting solution. This was transferred to a 250 ml conical flask for heating and stirred continuously. When the temperature reached 90 to 100°C, 10 ml of 38.8 mM sodium citrate was added as quickly as possible. After a few seconds the characteristic deep burgundy of gold nanosphere sols was attained. The sol was held at 90°C for a further 15 minutes after which the solution was removed from the heater. The resulting particles were of the order of 20 to 25 nm in diameter, Figure S11. The absorbance peak was at 526 nm in the as-prepared state, Figure S12. The method described yields a consistent and reproducible product. These sols are stable at least two years provided that they are stored in very clean containers. The presence of even tiny quantities of ions of other multivalent metals will cause the colloids to precipitate.

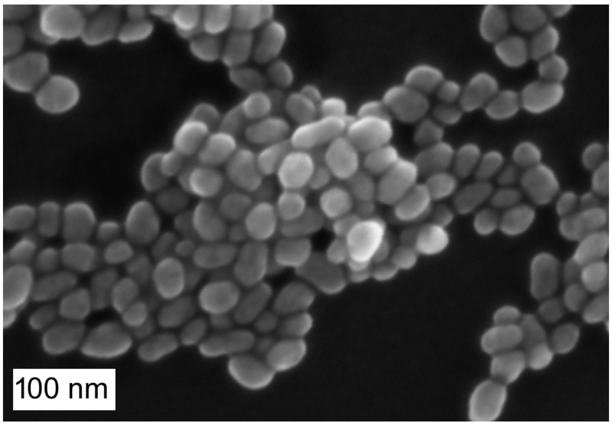

Supplementary Figure S11. Scanning electron microscope image of sample of Au NPs produced by our standardized 'citrate method' described above. (Image courtesy of Dr R. Wuhrer and Ms A. Dorrigan.)

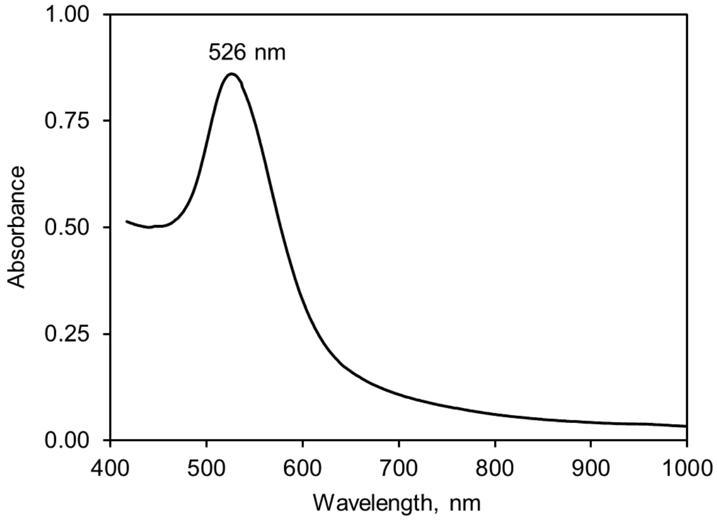

Supplementary Figure S12. UV-vis absorbance spectrum of Au NP colloid produced by our standardized ‘citrate method’ described above. (Data courtesy of Dr D. Pissuwan.)

Supplementary Table S1: TaqMan® gene expression assays probe information provided by the manufacture (Life Technology, CA, USA).

**Gene**	**NCBI gene references**	**FAM-labeled Probes (5'→ 3')**	**Assay ID**
18S	X03205.1	ACCGCAGCTAGGAATAATGGA	4319413E
ATGL	NM_025802.3	CCAAGACTGAATGGCTGGATGGCAA	Mm00503040_m1
CPT-1α	NM_031559.1	CCAGGAGAGTGCCAGGAGGTCATAG	Mm00550438_m1
GLUT4	NM_009204.2	GGCTCTGCTGCTGCTGGAACGGGTT	Mm00436615_m1
Leptin	NM_008493.3	TCACACACGCAGTCGGTATCCGCCA	Mm00434759_m1
TNFα	NM_001278601.1	GACCCTCACACTCAGATCATCTTCT	Mm00443258_m1
TLR-4	NM_021297.2	CCCTGCATAGAGGTAGTTCCTAATA	Mm00445273_m1

Supplementary Table S2. SYBR® Green expression assays primer information

**Marker**	**Forward primer sequence (5' → 3')**	**Reverse primer sequence (5' → 3')**
Adiponectin	CCACTTTCTCCTCATTTCTG	CTAGCTTCAGTTGTAGTAAC
SREBP-1c	AATAAATCTGCTGTCTTGCG	CCTTCAGTGATTTGCTTTTG
FAS	TGAATGCCTCAAATCTTAGC	TTTTAGCTTCCTGGATTGTC
FOXO1	AAACACATATTGAGCCACTG	TCTACTCTGTTTGAAGGAGG
F4/80	CTCCAAGCCTATTATCTATACC	CTTCCACAATCTCAC

Supplementary Table S3. Concentration of gold measured in the organs

Organ (μg Au/g)	Chow-C	HFD-C	HFD-LAu	HFD-HAu
**Fat**	0.1 ± 0.0	4.7 ± 1.4	7253 ± 3784*†	29883 ± 3154*†‡
**Liver**	2.4 ± 0.6	2.1 ± 0.6	128 ± 31*†	1792 ±1297*†
**Spleen**	5.1 ± 2.4	3.8 ±0.9	628 ± 148*†	1943 ± 196*†‡
**Kidney**	6.6 ± 1.8	1.3 ± 0.0	9.9 ± 1.7	23.8 ± 5.3*†‡
**Brain**	1.9 ± 1.0	1.1 ± 0.2	2.7 ± 1.6	12.1 ± 4.9*†‡
**Heart**	0.5 ± 0.2	0.3 ± 0.2	3.3 ± 2.2	2.1 ± 0.5

Data are expressed in mean ± S.E.M. Data were analyzed using one-way ANOVA, followed by *post hoc* Bonferroni tests. * *P<0.05 vs.* Chow-C; † *P<0.05 vs.* HFD-C; ‡ *P<0.05 vs.* HFD-LAu; *n*=3/group.

Supplementary Table S4: Effect of AuNP treatment on lipid metabolism in the 3T3-L1 adipocytes.

	**AD-C**	**AD-LAu**	**AD-HAu**
**Diameter of lipid droplets per cell (μm)**			
**1 h**	10.38 ± 0.62	9.68 ± 0.55	12.17 ± 0.61
**24 h**	6.01 ± 0.35	6.59 ± 0.38*	5.06 ± 0.25
**72 h**	9.08 ± 0.40	9.12 ± 0.42	7.60 ± 0.52*
**Supernatant triglyceride (mM)**			
**1 h**	0.27 ± 0.04	0.21 ± 0.04	0.27 ± 0.03
**24 h**	0.79 ± 0.10	0.64 ± 0.07	0.86 ± 0.12
**72 h**	2.74 ± 0.34	2.71 ± 0.36	2.86 ± 0.54

Data were analyzed using one-way ANOVA, followed by *post hoc* Bonferroni tests. Results are expressed as mean ± S.E.M. * $P<0.05$ *vs.* AD-C group; n=8.

Reference

1. Yamashita, A., *et al.* Macrophage‐adipocyte interaction: marked interleukin‐6 production by lipopolysaccharide. *Obesity* **15**, 2549-2552 (2007).

2. Zebisch, K., Voigt, V., Wabitsch, M. & Brandsch, M. Protocol for effective differentiation of 3T3-L1 cells to adipocytes. *Analytical Biochemistry* **425**, 88-90 (2012).
3. Suganami, T., Nishida, J. & Ogawa, Y. A paracrine loop between adipocytes and macrophages aggravates inflammatory changes role of free fatty acids and tumor necrosis factor α. *Arteriosclerosis, thrombosis, and vascular biology* **25**, 2062-2068 (2005).
4. Chou, J*., et al.* The therapeutic effect on bone mineral formation from biomimetic zinc containing tricalcium phosphate (ZnTCP) in zinc-deficient osteoporotic mice. *PLoS ONE* **8**, e71821 (2013).
